# Adaptive Integral Sliding Mode Control for Temperature Regulation in Gas-Phase Ethylene Polymerization Fluidized Bed Reactors

**DOI:** 10.3390/polym18040460

**Published:** 2026-02-11

**Authors:** Nayef Ghasem

**Affiliations:** Department of Chemical and Petroleum Engineering, United Arab Emirates University, Al Ain 15551, United Arab Emirates; nayef@uaeu.ac.ae

**Keywords:** gas-phase polymerization, fluidized bed reactor, temperature control, adaptive integral sliding mode control, nonlinear control, disturbance rejection, real-time control

## Abstract

Temperature regulation of gas-phase ethylene polymerization fluidized bed reactors (FBRs) is challenging due to strong nonlinearities, highly exothermic reaction kinetics, and frequent process disturbances. Conventional Proportional–Integral–Derivative (PID) control often exhibits limited robustness under such conditions, while advanced strategies such as Nonlinear Model Predictive Control (NMPC) may suffer from sensitivity to model mismatch and disturbances. In this study, an Adaptive Integral Sliding Mode Control (AISMC) strategy is proposed for temperature control of nonlinear gas-phase FBRs. The controller integrates adaptive gain adjustment with an integral sliding surface to improve disturbance rejection and steady-state accuracy while mitigating chattering. The performance of the proposed approach is evaluated through closed-loop simulations over an 18 h dynamic operating scenario involving multiple setpoint changes, catalyst activity variations, and feed flow disturbances. Simulation results demonstrate that AISMC achieves the best overall tracking performance, with a mean absolute error (MAE) of 0.092 K and the lowest maximum temperature deviation among the evaluated controllers. Compared to PID (MAE = 0.794 K) and conventional sliding mode control (MAE = 0.179 K), AISMC provides substantial improvements in transient and steady-state behaviors. In contrast, NMPC exhibits degraded tracking performance (MAE = 0.809 K) under the considered disturbance conditions. All controllers demonstrate sub-millisecond execution times; however, AISMC attains superior accuracy without excessive computational cost. These results indicate that AISMC offers an effective balance between robustness, accuracy, and real-time feasibility for industrial gas-phase polymerization reactors.

## 1. Introduction

**Figure 1:**
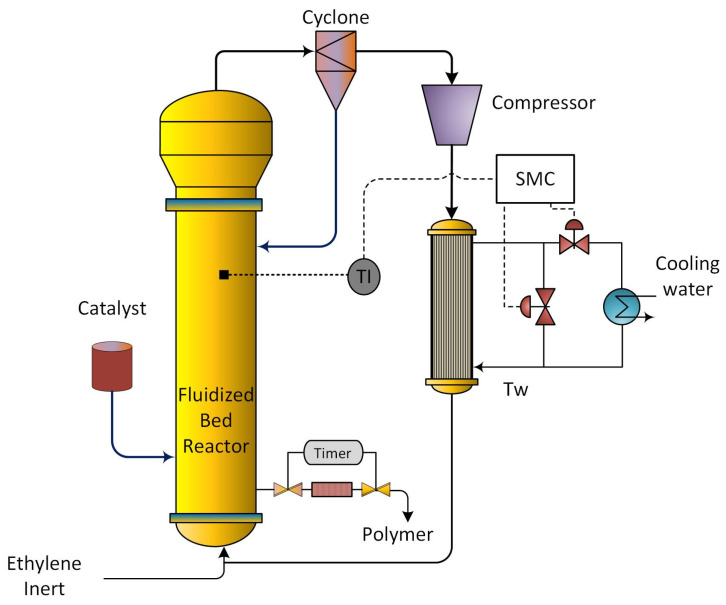
Schematic representation of ethylene polymerization in a fluidized bed reactor under sliding mode control (SMC).

Polyethylene production via ethylene polymerization is a cornerstone of modern polymer manufacturing, as polyethylene is one of the most widely used thermoplastics globally. Among the various reactor configurations, gas-phase fluidized bed reactors (FBRs) are preferred in industry because they provide excellent heat and mass transfer characteristics, scalability, and continuous operation [1,2]. A schematic diagram of the industrial FBR setup used in this study is shown in Figure 1. Within these reactors, ethylene and comonomers react with a solid catalyst fluidized by the upward flow of gas [3].

Traditionally, proportional–integral–derivative (PID) controllers dominate industrial practice due to their simplicity and low cost. However, extensive studies have demonstrated that PID controllers often fail to handle the nonlinear dynamics, time-varying process conditions, and disturbance sensitivities typical of FBRs. For example, recent studies [4,5,6] have demonstrated that catalyst structure and active-site design play a critical role in controlling ethylene polymerization reactivity and performance. Salau et al. [7] and Luo et al. [8] reported bifurcation-induced oscillations and potential instability during closed-loop PID control when feed composition or catalyst activity changes. These findings have motivated the development of advanced control techniques capable of handling these nonlinearities [9,10,11]. The research by Dadebo and Bell [12] showed enhanced temperature tracking performance through the use of external heat exchanger dynamics and nonlinear error trajectory control. Model Predictive Control (MPC) has emerged as a strong candidate for advanced polymerization process control due to its constraint-handling capabilities and superior multivariable regulation performance. Early studies on the nonlinear control of gas-phase polymerization reactors laid the groundwork for advanced strategies. Sato et al. [13] proposed a rigorous nonlinear reactor model with trajectory-based control, while McAuley [14] introduced an extended Kalman filter coupled with nonlinear feedback to enhance real-time property control. However, these early approaches relied heavily on precise model knowledge and were prone to performance degradation under parameter uncertainties and operational disturbances, motivating the exploration of more robust techniques such as model predictive control and, more recently, sliding mode control [15].

Seki et al. [16] and Muhammad et al. [17] successfully applied state-space MPC to polyethylene reactors, achieving faster transitions and better disturbance rejection compared to PID. Liu and Sharma [18,19] further demonstrated the role of hybrid data-driven models and MPC in enhancing prediction accuracy and control efficiency in polyolefin manufacturing. Fuzzy logic and neuro-fuzzy controllers have also been explored extensively.

Ghasem [15] introduced a fuzzy-PI controller for polyethylene FBRs that improved transient response, while Abbasi et al. [20] and Salahuddin et al. [21] designed hybrid ANFIS and fuzzy-GMC controllers, achieving enhanced setpoint tracking and disturbance rejection. Ali [22] combined fuzzy logic with a sliding mode observer to estimate unmeasured states such as monomer concentration and Melt Flow Index (MFI), improving estimation accuracy under uncertain operating conditions.

Kazerooni et al. [23] validated a two-reactor model for Low-Density Polyethylene (LDPE) production using Ziegler–Natta kinetics, while Li et al. [24] integrated kinetic mechanisms with property prediction models using the method of moments to accurately forecast MFI and molecular weight distribution. Additionally, Jani et al. [25] addressed the lack of real-time measurements in quality control by developing soft sensors using segmented Gaussian process regression and ensemble learning, significantly improving the prediction of MFI, density, and particle diameter.

Despite these developments, many advanced controllers face practical challenges, including high computational complexity, model sensitivity, and difficulty in real-time implementation. Sliding Mode Control (SMC) offers a compelling alternative. As a nonlinear robust control method, SMC drives system trajectories onto a predefined sliding surface and maintains them there using a discontinuous control law. It is highly robust to bounded model uncertainties and external disturbances, and it guarantees finite-time convergence, making it especially suitable for the highly dynamic and nonlinear environment of ethylene FBRs [26].

Recent advancements in control strategies for continuous polymerization reactors have gained significant attention. Maaruf et al. [27] proposed an AI-based adaptive control framework to handle input dead-zone nonlinearities, achieving improved tracking performance and robustness under uncertain operating conditions. Mahmoud et al. [28] developed a neuro-adaptive fast terminal sliding mode controller capable of compensating for unknown disturbances while ensuring fast convergence and high control accuracy. Additionally, Maaruf et al. [29] presented an adaptive control strategy that dynamically adjusts controller parameters to improve stability and performance in continuous polymerization processes. Recent advances in sliding mode control (SMC) have demonstrated strong potential for handling nonlinear, time-delayed, and uncertain systems in various engineering fields. For example, Zuo et al. [30] proposed a fuzzy sliding mode controller with delay compensation, while Zhou et al. [31] developed an enhanced hyperbolic SMC strategy to improve transient performance and reduce chattering in UAV systems. Similarly, Vimala Devi et al. [32] conducted a comparative analysis of SMC and model predictive control (MPC), highlighting the superior robustness of SMC under rapid disturbances. Additionally, Shang and Wang [33] introduced an integral sliding mode control framework based on an improved exponential reaching law, demonstrating how integral surfaces can effectively eliminate steady-state errors and enhance robustness. Gao et al. [34] proposed a delay-compensated SMC design with prescribed-time convergence, which is particularly relevant given the delayed thermal dynamics of polymerization reactors.

These recent studies highlight the potential of intelligent and adaptive control methods for addressing complex nonlinear dynamics. While advanced SMC strategies have been widely investigated for chemical process control in general, their application to gas-phase polyethylene polymerization fluidized bed reactors, particularly using adaptive integral sliding mode control, remains relatively limited. The wording has been revised accordingly to more accurately reflect the existing literature.

To bridge this gap, this work proposes an Adaptive Integral Sliding Mode Control (AISMC) strategy that combines an integral sliding surface with an online adaptive gain adjustment mechanism. Unlike conventional integral or adaptive SMC approaches with fixed or heuristically tuned gains, the proposed AISMC dynamically updates the switching gain based on the magnitude of the sliding surface, allowing improved disturbance rejection while reducing steady-state error and chattering. This design enables performance comparable to nonlinear model predictive control (NMPC) in terms of temperature tracking accuracy, while maintaining significantly lower computational complexity.

Table 1 summarizes the main control strategies that have been reported in the literature for gas-phase polyethylene fluidized bed reactors with their main advantages, disadvantages, prediction accuracy, and application to this problem. Proportional–Integral–Derivative (PID) controllers are usually preferred due to their ready availability and cheapness of implementation, though they demonstrate very poor performance under nonlinear and time-varying conditions. Better choices include multivariable model predictive control (MPC) systems and hybrid data-driven systems because they possess high prediction accuracies and abilities to control more than one variable, but they are also associated with the drawbacks of even more significant computational requirements and an explicit need for accurate models or large amounts of data. Intelligent systems such as fuzzy logic control and neuro-fuzzy systems improve adaptability and transient performance but introduce new tuning and training problems. Sliding mode control, together with its adaptive versions, presents great robustness and finite-time convergence. Therefore, they are most appropriate in such highly nonlinear and uncertain environments as characterized by polymerization reactors. It is against this background that integral sliding mode control with adaptation has been proposed here.

## 2. Mathematical Model

The gas phase is assumed to be perfectly mixed, with no axial or radial gradients in temperature or composition. Only the main polymerization reaction is considered, and side reactions and byproduct formation are neglected. All gaseous species are assumed to follow the ideal gas law. Physical properties such as the heat capacities, the heat transfer coefficient, and the reaction enthalpy are taken to be constant. The heat removal unit is represented as a first-order dynamic response, with a constant time constant. The pressure is uniform in the reactor, and the axial pressure drop is negligible. The polymerization kinetics is given by an Arrhenius-type expression with a constant activation energy and pre-exponential factor [15].

The effective heat capacity of the gas-phase mixture:

(1) $$C_{pg}\;=\frac{\left(M_{1}\;\times \;C_{p},M1\;+\;In\;\times \;C_{p},In\right)}{\left(M_{1}+\;In\right)}$$

The overall heat transfer coefficient term based on gas and coolant sides:

(2) $$\gamma \;=\;A_{U}\;*\;\left(\frac{1}{\left(F_{g}\times \;C_{pg}\right)}+\;\frac{1}{\left(F_{w}\;\times \;C_{pw}\right)}\right)$$

The ratio of heat transport capacity between gas and coolant flows:

(3) $$\eta \;=\frac{\left(F_{g}\;\times \;C_{pg}\right)}{\left(F_{w}\;\times \;C_{p,w}\right)}$$

The outlet gas temperature after heat exchange:

(4) $$T_{gos}\;=\frac{\left(T_{w}\;\times \;\left(1\;-\;exp\left(-\gamma \right)\right)-\;T\;\times \;\left(1\;-\eta \right)\right)}{\left(\eta \;-\;exp\left(-\gamma \right)\right)}\;$$

Heat removal from the reactor through the gas stream:

(5) $$Q_{ss}\;=\;F_{g}\;\times \;C_{pg}\;\times \;(T\;-\;T_{gos})$$

Temperature-dependent reaction rate constant (Arrhenius form):

(6) $$k_{pi}\;=\;k_{p0}\;\exp\left[-\frac{E_{a}}{R}\left(\frac{1}{T}\;-\;\frac{1}{T_{ref}}\right)\right]$$

Monomer consumption rate based on concentration and polymer fraction:

(7) $$R_{M1}\;=\;M_{1}\times \;k_{pi}\;\times \;Y$$

Polymer production rate in mass terms:

(8) $$O_{p}\;=\;R_{M1}\;*\;M_{w1}$$

Vent flow rate as a function of pressure differential:

(9) $$b_{t}\;=\;v_{p}\;\times \;C_{v}\;\times \sqrt{P_{tot}\;-\;P_{v}}\;$$

The following PI control law is implemented to regulate ethylene pressure as a function of changes in the ethylene composition rate. The flow rate of monomer ($F_{M}$):

(10) $$F_{M}=F_{M0}+K_{p}\left[e(t)+\frac{1}{\tau_{I}}\int_{0}^{t}e(t)dt\right]$$

where $F_{M0}$ is the feed rate when the controller is turned off, and $K_{p}$ and $\tau_{I}$ are proportional and integral gains, respectively. The error $e\left(t\right)$ is between the ethylene partial pressure setpoint and the actual ethylene partial pressure. The ideal gas law is used to measure the actual partial pressure of ethylene, $P_{M}=C_{M}RT$, where CM is the ethylene concentration.

**Energy Balances**

Enthalpy input from monomer and inert feeds:

(11) $$H_{f}\;=\;(F_{M1}\;\times \;C_{p,M1}\;+\;F_{In}\;\times \;C_{p,In})\;\times (T_{feed}\;-\;T_{ref})$$

Enthalpy is carried out by gas and vent flows:

(12) $$H_{go}\;=\;(F_{g}\;+\;b_{t})\;\times \;C_{pg}\;\times \;(T\;-\;T_{ref})$$

Net gas-phase enthalpy after accounting for heat removal:

(13) $$H_{gi}\;=\;H_{go}\;-\;Q_{ss}$$

Equation (13) represents the net gas-phase enthalpy after accounting for steady-state heat removal $Q_{ss}$.

Heat generated by the exothermic polymerization reaction:

(14) $$H_{r}\;=\;-∆H\;\times \;M_{w1}\;\times \;R_{M1}$$

**Model Differential Equations**

Dynamics of heat removal system with first-order response:

(15) $$\frac{dQ}{dt}\;=\frac{\left(Q_{ss}\;-\;Q\right)}{\tau }$$

Mass balance of inert gas in the reactor:

(16) $$\frac{dIn}{dt}\;=\frac{\left(F_{In}\;-\frac{\left(In\;\times \;b_{t}\right)}{\left(In\;+\;M_{1}\right)}\right)}{V_{g}}\;$$

Mass balance of monomers in the reactor:

(17) $$\frac{dM_{1}}{dt}\;=\frac{\left(F_{M1}\;-\frac{\left(M_{1}\;\times \;b_{t}\right)}{\left(In\;+\;M_{1}\right)}-\;R_{M1}\right)}{V_{g}}$$

Dynamics of polymer mass fraction in the reactor:

(18) $$\frac{dY}{dt}\;=\;F_{c}\;\times \;a_{c}\;-\;k_{d}\;\times \;Y\;-\;Y\;\times \;\frac{O_{p}}{B_{w}}$$

Substituting Equation (13) into the reactor energy balance yields the final temperature evolution equation used in the simulations. Reactor temperature changes considering all energy sources and sinks:

(19) $$\frac{dT}{dt}\;=\frac{\left(H_{f}\;+\;H_{gi}\;-\;H_{go}+H_{r}\;-O_{p}\;\times C_{p,pol}\;\times \;\left(T\;-\;T_{ref}\right)\right)}{\left(M_{r}\;\times \;C_{pr}\;+\;B_{w}\;\times \;C_{p,pol}\right)}$$

Integral error accumulation for pressure control logic:

(20) $$\frac{dIE}{dt}\;=\;P_{sp}\;-\;M_{1}\;\times R_{1}\;\times \;T$$

All variables, parameters, units, and physical meanings are summarized in Table 2, ensuring consistency and reproducibility of the proposed model.

## 3. Sliding Mode Control Design

The Sliding Mode Control (SMC) strategy is adopted for temperature regulation in the fluidized bed reactor (FBR) due to its strong robustness against nonlinearities, model uncertainties, and external disturbances. The primary objective is to maintain the reactor temperature at the desired setpoint while ensuring stable operation and consistent product quality, even under uncertain process conditions [22,35,36,37].

### 3.1. Sliding Surface

The sliding surface is defined as

(21) $$s(t)=\dot{e}(t)+\lambda e(t)$$

where $e(t)=T(t)-T_{\mathrm{set}}$ is the temperature tracking error and λ>0 is the sliding surface parameter. The parameter λ governs the convergence speed of the tracking error dynamics. A larger λ leads to faster error convergence but may increase control effort and chattering, while a smaller λ results in smoother control action at the expense of slower response. In this study, λ was selected based on the closed-loop thermal time constant of the reactor, dominant pole placement of the linearized temperature dynamics, and the requirement that the sliding surface dynamics $\left(\dot{e}+\lambda e=0\right)$ remain sufficiently faster than the reactor’s natural dynamics to ensure effective disturbance rejection. Based on these considerations, λ was chosen as 0.8, and a sensitivity analysis was performed over the range 0.5≤λ≤1.2, which was found to provide a good trade-off between fast convergence and smooth control action.

### 3.2. Control Law

The control input is designed as:

(22) u(t)=−K sign(s(t))

where:

u(t) is the control action (e.g., coolant flow rate adjustment),

K is the control gain,

sign (·) denotes the signum function.

The signum function determines the sign of its argument and is defined as:

(23) $$sign\left(s\right)=\left\{\begin{array}{c} +1,\;s>0\;\\ 0,\;s=0\\ -1,\;s<0 \end{array}\right.$$

This function ensures that the control law forces the system states toward the sliding surface s = 0, making the control discontinuous to achieve fast convergence.

### 3.3. Boundary Layer to Reduce Chattering

To mitigate chattering caused by the discontinuous sign function, a smooth approximation is applied using a saturation function:

(24) $$u(t)\;=\;-K\;sat\left(\frac{s\left(t\right)}{\varphi }\right)\;$$

where φ is the boundary layer thickness and sat (·) ensures a continuous control signal. A properly selected φ effectively reduces high-frequency oscillations without compromising tracking performance.

### 3.4. Difference Between Conventional SMC and Integral SMC

In conventional SMC, the sliding surface depends only on the error and its derivative:

(25) s(t) = ė(t) + λe(t)

However, Integral Sliding Mode Control (ISMC) introduces an additional integral term to improve robustness and eliminate steady-state error:

(26) $$s(t)\;=\;ė(t)\;+\;\lambda e(t)\;+\;\gamma \int_{0}^{t}e(\tau )d\tau$$

where γ > 0 is the integral gain. Advantages of ISMC over Conventional SMC:

- Eliminates steady-state error caused by model uncertainties,
- Enhances disturbance rejection capability,
- Produces smoother control effort and reduces chattering,
- Ensures the system starts on the sliding surface from t = 0, improving robustness.

### 3.5. Stability Analysis

To ensure closed-loop stability, Lyapunov’s direct method is applied with the candidate function:

(27) $$V\left(s\right)=\frac{1}{2}\;s^{2}$$

Taking its derivative along the trajectories of the system:

(28) $$V^{˙}\;=\;s\;s˙\;=\;-K|s|\;\leq \;0$$

Since V^˙^ ≤ 0, the system is globally asymptotically stable under the proposed SMC framework.

### 3.6. Simulation Setup and Implementation

All simulations for the proposed Adaptive Integral Sliding Mode Control (AISMC) and Nonlinear Model Predictive Control (NMPC) strategies were carried out using Python 3.11 on a workstation running Windows 11 Pro (64-bit), equipped with an Intel^®^ Core™ i9-12900K CPU @ 3.19 GHz and 128 GB of RAM. The large memory and high processing speed of this workstation ensured efficient handling of the complex dynamic reactor model and real-time control computations. The reactor model was integrated using the Radau method in solve_ivp with adaptive internal step sizes and tolerances rtol = 1 × 10^−6^ and atol = 1 × 10^−6^. The control laws (PID, SMC, AISMC, and NMPC) were executed with a sampling time of Δt = 5 s over the 18 h simulation horizon.

For control implementation, the NMPC algorithm was developed using CasADi 3.6.3 due to its efficiency in solving large-scale nonlinear optimization problems in real time. The AISMC strategy, on the other hand, was implemented using NumPy 2.1.3, and custom Python 3.11.11, control scripts to allow full flexibility and precise implementation of the proposed sliding mode algorithm. Under these settings, the average computation time per control step was approximately 2.4 ms for AISMC and 18.7 ms for NMPC, demonstrating that AISMC offers faster real-time performance while NMPC provides higher computational complexity due to the repeated optimization steps. These implementation details and computational specifications are provided to ensure full reproducibility of the reported results.

## 4. Results and Discussion

This section presents the outcomes of the simulation study, which includes both open-loop sensitivity analysis and closed-loop control using SMC. The sensitivity analysis was conducted to identify the influence of key process variables, such as catalyst feed rate, coolant temperature, and gas flow rate, on reactor temperature. Subsequently, SMC was implemented to regulate the reactor temperature under dynamic conditions, demonstrating effective setpoint tracking and robustness against disturbances, including catalyst and gas feed rate variations. The comparative results highlight the superior performance of SMC over conventional PID control in handling nonlinear dynamics and maintaining thermal stability.

### 4.1. Open-Loop Sensitivity Analysis

The open-loop sensitivity analysis was conducted to study how essential process parameters affected reactor temperature when active control was not implemented. The study examined one parameter at a time by adjusting the catalyst feed rate, the coolant temperature, and the gas flow rate while keeping all other parameters constant. The simulation demonstrated that coolant temperature and catalyst feed rate were the primary factors affecting thermal behavior, with gas flow rate variations having only a moderate impact. The study reveals how the reactor responds to changes over time and demonstrates why precise management of heat inputs is essential for maintaining stable reactor operations.

**Figure 2:**
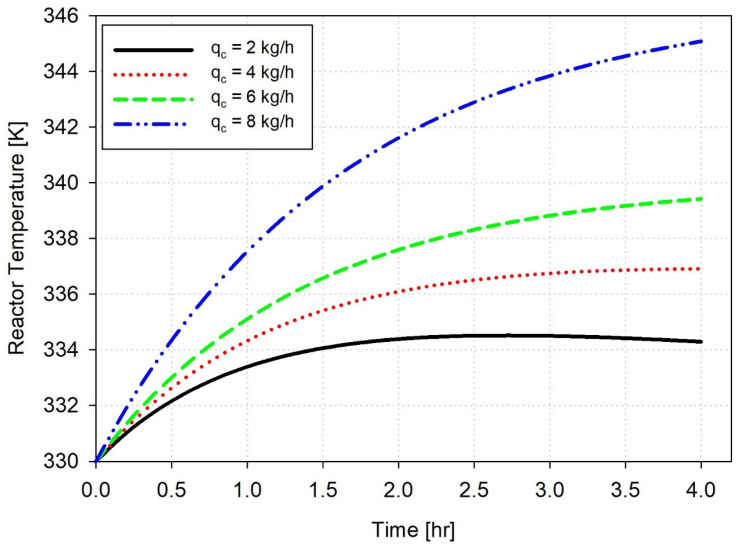
Open-Loop reactor temperature response at different catalyst feed rates and constant gas inlet temperature (T_0_ = 330 K).

Figure 2 illustrates the open-loop reactor temperature profiles over a 4 h operation period for four different catalyst feed rates: 2, 4, 6, and 8 kg/h. The reactor begins operation at 330 K without using feedback control mechanisms. Simulation prediction shows how the ethylene polymerization process reacts thermally to various catalyst injection rates. Due to accelerated polymerization reactions and heat release, the reactor temperature rises quickly and continues to increase at 6 and 8 kg/h feed rates. Lower feed rates, such as 2 kg/h, led to a slower temperature increase, stabilizing or decreasing over time because heat production matches heat dissipation. When the catalyst feed rate is set to 4 kg/h, the system reaches a near-constant temperature of 338 K, which suggests a quasi-steady state condition. As shown in Figure 2, increasing the catalyst feed rate results in a higher reactor temperature due to enhanced reaction kinetics. For $q_{c}$ = 8 kg/h, the temperature rise is noticeably steeper during the initial transient. This behavior is attributed to the nonlinear dependence of the heat generation rate on catalyst loading, which causes a rapid increase in exothermic reaction heat at higher $q_{c}$.

The observed thermal behavior patterns show that the reactor dynamics are nonlinear, which require active temperature control mechanisms when using elevated catalyst amounts [38]. Without temperature control systems to manage reactor conditions, high rates of catalyst injections produce excessive exothermic polymerization heat, which exceeds the reactor’s natural cooling abilities. The lack of thermal equilibrium produces fast, uncontrollable temperature spikes known as thermal runaways that have the potential to harm equipment, activate catalysts, or degrade product quality. Low catalyst feed rates reduce thermal instability risks but result in inadequate monomer conversion and lower polymer production rates, which reduce overall efficiency and economic viability. Precise catalyst dosing must work in conjunction with dependable temperature regulation to operate reactors safely and efficiently while achieving high performance.

**Figure 3:**
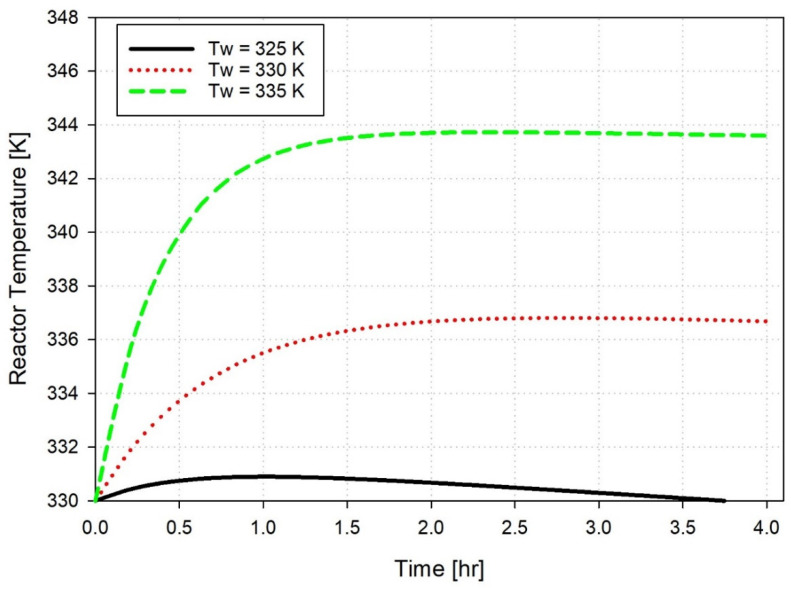
Effect of coolant temperature near 330 K on reactor temperature at fixed catalyst feed rate (qc = 3.5 kg/h).

Variations in coolant inlet temperature (T_w_) demonstrate the gas-phase ethylene polymerization reactor’s strong thermal sensitivity, as shown in Figure 3, while maintaining a constant catalyst feed rate of 3.5 kg/h. The simulation reveals a consistent and physically intuitive trend: when T_w_ rises from 325 K to 335 K, the reactor temperature also increases. This behavior occurs because the heat transfer rate declines with the reduced temperature gradient between the reactor and the cooling medium. The reactor holds onto a greater portion of the heat produced by the exothermic polymerization reaction. The reactor reaches its peak temperature sooner and attains a higher steady-state temperature when T_w_ is increased. The data demonstrates an accelerated buildup of thermal energy and reduced efficiency in removing heat from the system. These conditions create operational hazards, resulting in thermal runaways, catalyst deactivation, or improper molecular weight distribution in the polymer product. The research demonstrates that exact and responsive control of T_w_ is essential for thermal stability maintenance and safety enhancement while optimizing polymerization kinetics for superior product quality. Similar results were reported in a study that employed computational fluid dynamics (CFD) to examine the impact of coolant inlet temperature variations on the thermal behavior of polymerization reactors. The study found that even slight increases in T_w_ significantly reduce heat removal efficiency, leading to higher reactor temperatures and an increased risk of thermal instability, highlighting the importance of precise coolant temperature control [39]. The selection of input parameters for simulation was guided by practical operating considerations and the objectives of each analysis. The catalyst feed rate was varied over a wide range (2–8 kg/h) to capture the nonlinear influence of catalyst loading on reaction heat generation and temperature dynamics. For controller performance evaluation under coolant disturbances, a nominal catalyst feed rate of 3.5 kg/h was selected to represent typical operating conditions and to decouple the effects of catalyst loading from coolant temperature variations. The coolant temperature varied within a ±5 K range, consistent with realistic industrial cooling constraints, which was sufficient to highlight the system’s sensitivity and controller robustness without exceeding safe operating limits.

**Figure 4:**
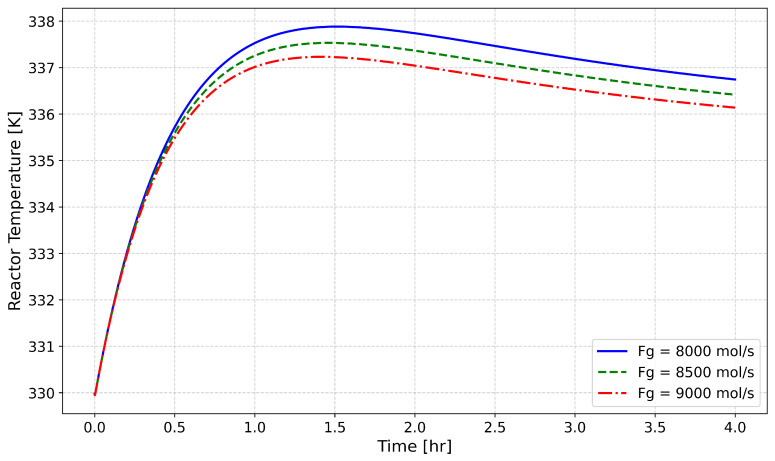
Effect of Gas Flow Rate Near 8500 mol/s on Reactor Temperature (qc = 3.5 kg/h).

Figure 4 demonstrates how different feed gas flow rates (F_g_) affect reactor temperature profiles during open-loop operation while keeping the catalyst feed rate fixed. The peak and steady-state reactor temperatures experience a uniform decrease when the F_g_ flow rate is increased from 8000 mol/s to 9000 mol/s. Two simultaneous processes lead to the observed thermal behavior. The elevated flow rate leads to better convective heat transfer inside the reactor, which enables more efficient heat removal from the exothermic polymerization reaction. The increased flow rate leads to a dilution effect on the ethylene monomer concentration, resulting in a diminished reaction rate and a decreased thermal output. In industrial environments, slight temperature differences result in significant operational effects because reactor stability, catalyst life, and polymer quality depend heavily on thermal stability. The research conducted by Ghasem [11] confirms through population balance modeling that higher gas flow rates result in better thermal control by increasing heat dissipation and decreasing the intensity of localized reactions. Precise F_g_ management is essential to keep thermal stability and process safety in check while producing uniform polymer characteristics within gas-phase ethylene polymerization reactors.

### 4.2. Implementation of Closed-Loop Control

Sliding Mode Control (SMC) is a nonlinear control methodology that robustly forces system states onto a predetermined sliding surface. It maintains them there, even when confronted with model inaccuracies and external disruptions. This control strategy adjusts its input based on whether the tracking error is positive or negative, allowing for both rapid convergence and resilience to parameter variations once the sliding surface is reached. SMC effectively replaces traditional controllers in fluidized bed reactor chemical processes characterized by nonlinear behaviors and disturbances by providing improved disturbance response and strong stability assurances. To enhance the controller’s responsiveness and robustness, the adaptive gain in the AISMC is dynamically adjusted based on the temperature error signal and its rate of change. Specifically, the gain increases when the error magnitude or its derivative exceeds predefined thresholds, allowing the controller to react more aggressively to disturbances. This mechanism ensures stability and improves tracking performance under varying operating conditions.

**Figure 5:**
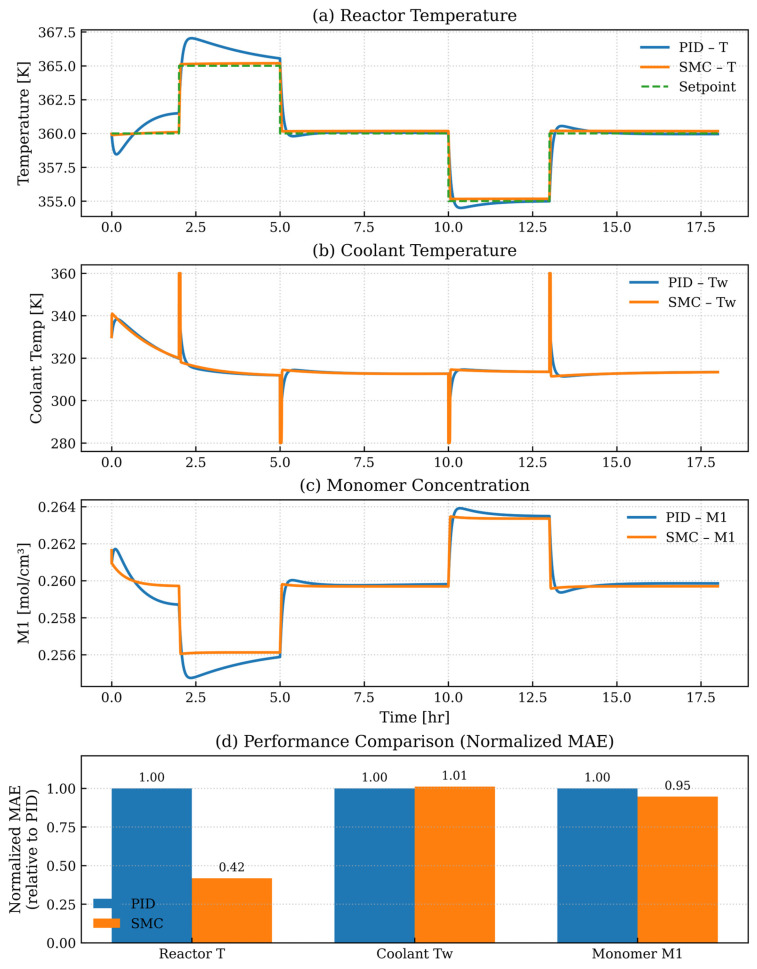
Dynamic response comparison between PID and Sliding Mode Control (SMC) for reactor temperature control. (**a**) Reactor temperature under varying setpoint conditions, (**b**) corresponding coolant temperature adjustments, (**c**) resulting in monomer concentration over time, and (**d**) quantitative performance comparison based on the mean absolute error (MAE), normalized with respect to the PID controller.

Figure 5 compares PID and Sliding Mode Control (SMC) strategies for controlling temperature in an ethylene polymerization fluidized bed reactor by examining reactor temperature, coolant temperature, and monomer concentration. Figure 5a illustrates that the PID controller exhibits significant overshoot and delayed settling times when the setpoint changes abruptly to 2.5, 5, and 10 h. At the same time, SMC reaches the desired setpoint more quickly with less overshoot and better tracking accuracy. The better performance demonstrates how SMC naturally withstands model uncertainties and external disturbances. Figure 5b illustrates how SMC provides smoother and more efficient heat exchange control through rapid yet stable coolant adjustments, contrasting the erratic responses of PID regulation. Stable coolant dynamics are crucial in minimizing thermal stress within the reactor system. Unlike PID control, the SMC regulation technique enables monomer concentration (Figure 5c), which reacts to thermal dynamics and impacts polymerization kinetics, to maintain stability with faster stabilization and minimized transient deviations. SMC demonstrates top control performance by improving setpoint tracking accuracy, disturbance rejection, and process stability across all essential reactor variables, confirming its reliability for nonlinear and thermally sensitive polymerization systems. While Figure 5a–c qualitatively illustrate the dynamic responses of the PID and SMC controllers, Figure 5d shows a normalized MAE-based comparison between the PID and SMC controllers. The SMC demonstrates significantly improved reactor temperature tracking, with smaller improvements in monomer concentration and comparable coolant temperature behavior.

**Figure 6:**
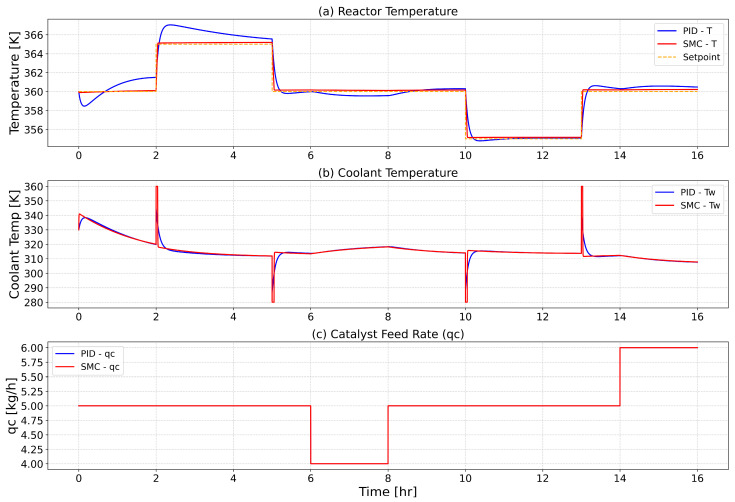
Comparison of PID and Sliding Mode Control (SMC) under catalyst feed disturbance. (**a**) Reactor temperature, (**b**) coolant temperature, and (**c**) disturbance in catalyst feed rate (qc) over time.

Figure 6 assesses the comparative robustness of PID and SMC strategies in regulating reactor temperature within a fluidized bed ethylene polymerization reactor, particularly under conditions of catalyst feed disturbances. In subplot (a), the reactor temperature trajectories reveal that the conventional PID controller struggles to maintain accurate tracking during transient phases, displaying significant overshoot and prolonged settling times, especially following the disturbance events introduced at 6 and 14 h. These deviations highlight the limitations of linear PID control in adapting to abrupt process variations in highly nonlinear reactor environments. Conversely, the SMC controller demonstrates a markedly improved performance, exhibiting minimal overshoot, faster convergence to the setpoint, and sustained stability throughout the disturbance periods. Subplot (b) further supports this conclusion by illustrating the corresponding coolant temperature actuation profiles. The PID controller reacts with sharp and erratic changes in coolant flow, indicative of reactive control effort and limited robustness. In contrast, the SMC strategy adjusts the coolant temperature more gradually and flexibly, ensuring smoother thermal compensation with reduced control effort. Subplot (c) displays the manipulated catalyst feed rate, which is subjected to deliberate step reductions and increases, emulating realistic disturbances in catalyst supply. Despite identical disturbance profiles applied in both cases, only the SMC controller maintains stable reactor operation without significant deviation from the temperature setpoint. These results underscore the superior disturbance rejection capabilities and robustness of SMC in managing nonlinearities, actuator constraints, and uncertainties inherent in gas-phase polymerization processes.

**Figure 7:**
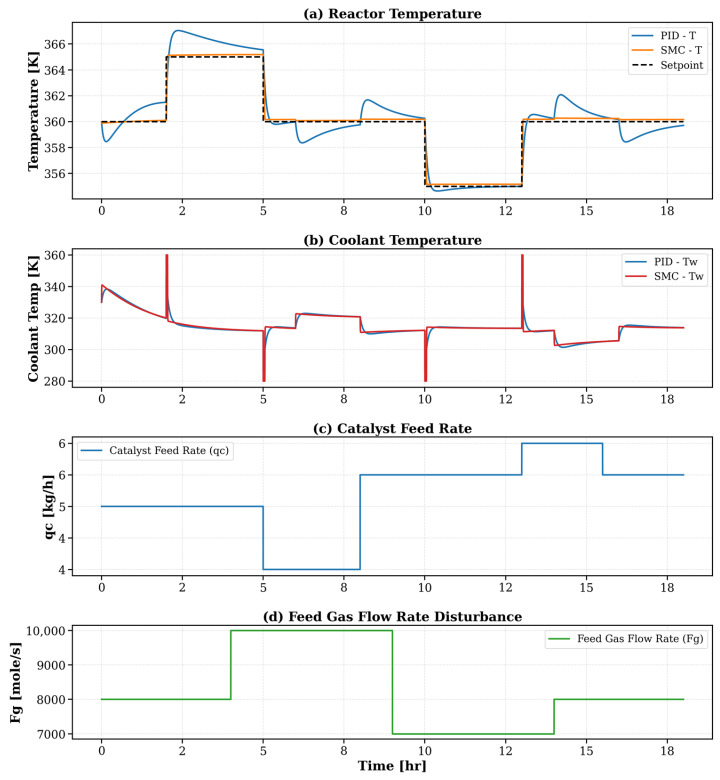
Dynamic response of PID and Sliding Mode Controllers under catalyst and feed gas flow disturbances. (**a**) Reactor temperature, (**b**) coolant temperature, (**c**) catalyst feed rate (qc), and (**d**) feed gas flow rate (F_g_).

Figure 7 presents a comprehensive assessment of the performance of PID and SMC strategies under realistic disturbance scenarios in a fluidized bed ethylene polymerization reactor. The analysis spans four key variables to capture process behavior and control effectiveness. In subplot (a), the reactor temperature response highlights the SMC controller’s superior setpoint tracking and robustness. The PID controller suffers from noticeable overshoot during the feed gas increase (4–6 h) and undershoot during the reduction phase (12–14 h), indicating a limited ability to reject step disturbances. Conversely, SMC maintains tighter control and faster recovery, minimizing deviation and oscillation around the setpoint. Subplot (b) shows the coolant temperature adjustments required by each controller. The PID controller responds to disturbances with abrupt and often excessive swings in coolant temperature, reflecting a reactive strategy prone to overcompensation. In contrast, the SMC controller modulates Tw more smoothly and anticipates the system’s nonlinear dynamics, contributing to its superior thermal regulation performance. Subplot (c) confirms the catalyst feed rate (qc) variation over time, which acts as a structured disturbance. This variation was uniformly applied in both control scenarios to simulate fluctuations in catalyst injections during operation. The ability of the SMC to maintain reactor stability in the presence of this variable reinforces its disturbance rejection capability. Subplot (d) explicitly depicts the feed gas flow rate (F_g_) disturbance, where a +20% step change was introduced between 4 and 6 h, followed by a −20% step between 12 and 14 h. This controlled perturbation tests each controller’s ability to adapt to load changes. The SMC controller demonstrated resilience to these disturbances, while the PID controller exhibited delayed and imprecise compensatory behavior. This figure validates that Sliding Mode Control provides significantly enhanced robustness, better setpoint adherence, and smoother actuation under complex dynamic conditions, making it a compelling choice for advanced temperature control in industrial polymerization reactors.

The quantitative error analysis above demonstrates how PID and Sliding Mode Control (SMC) strategies compare in terms of control performance. The SMC controller achieves a mean absolute error of 0.177 K, representing a substantial improvement over the PID controller’s error of 0.567 K. During the simulation period, the SMC controller demonstrated superior steady-state performance by keeping the reactor temperature closer to the desired setpoint on average. The low standard deviation in the error measure of variability supports the consistent performance of the SMC response. The SMC displays superior control stability and accuracy under dynamic conditions, as indicated by its standard deviation of 0.268 K, which is less than half of PID’s 0.599 K. The comparable maximum error values between SMC and PID controllers show that both face similar-sized transient disturbances. The SMC demonstrates superior average and variance performance, showing its faster response to disturbances and quicker system stabilization. The compiled metrics show that SMC surpasses PID concerning both precision and stability, which makes it the superior control choice for temperature control in nonlinear environments with disturbances.

### 4.3. Comparative Analysis

**Figure 8:**
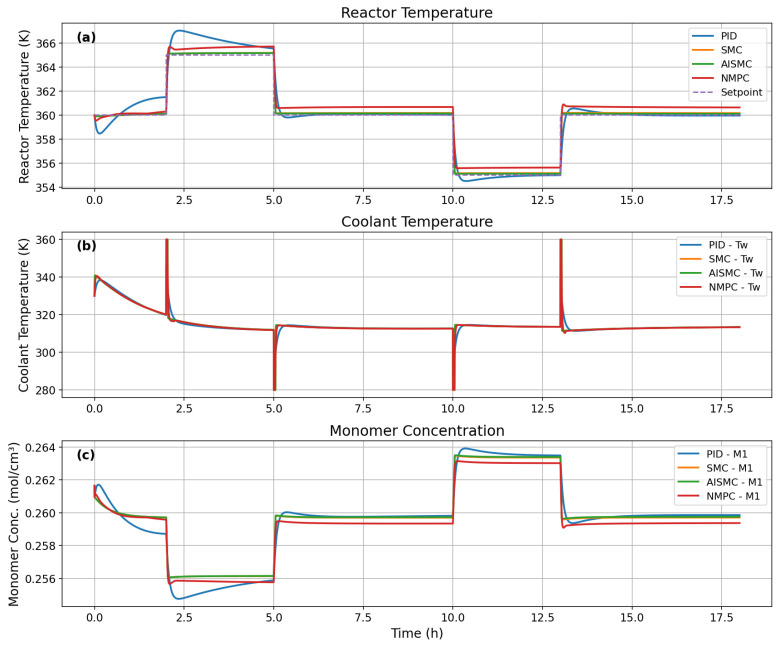
Dynamic performance comparison of four control strategies, PID, SMC, AISMC, and NMPC. (**a**) reactor temperature response, (**b**) coolant temperature response, and (**c**) monomer concentration response.

Figure 8 compares the closed-loop performance of the PID, SMC, AISMC, and NMPC controllers under multiple reactor temperature setpoint changes. The PID controller exhibits noticeable overshoot and slower settling, particularly during step changes, indicating limited robustness to nonlinear reactor dynamics. In contrast, SMC and AISMC significantly improve tracking performance, with reduced overshoot and faster convergence. AISMC provides smoother transitions than SMC due to adaptive gain adjustment. Although NMPC yields smooth reactor temperature profiles, a small steady-state offset from the setpoint is observed during some operating periods. The coolant temperature responses (Figure 8b) reveal that PID reacts aggressively to disturbances, while SMC and AISMC introduce short-duration control spikes inherent to sliding mode control. NMPC produces anticipative but computationally intensive control actions. The monomer concentration profiles (Figure 8c) further confirm that improved temperature regulation leads to reduced concentration deviations, with AISMC and NMPC achieving the most stable behavior.

These observations are quantitatively supported by Table 3. PID exhibits higher MAE and standard deviation, whereas SMC and AISMC substantially reduce tracking errors. AISMC achieves the lowest MAE, confirming its superior accuracy and robustness. Despite its smooth response, NMPC shows higher MAE and maximum error, consistent with the steady-state offset observed in Figure 8 and incurs the highest computational cost. Overall, AISMC offers the best compromise between tracking performance and real-time feasibility for polymerization reactor control.

### 4.4. Robustness Analysis Under Industrial Disturbances

To comprehensively evaluate the robustness of the proposed control strategies, additional simulations were performed under two common industrial disturbance scenarios:

1. Catalyst Deactivation/Activation: catalyst activity was reduced by 20% between 6 and 8 h and increased by 20% between 14 and 16 h to simulate typical catalyst aging and regeneration cycles.
2. Feed Composition Fluctuations: the ethylene feed concentration varied sinusoidally by ±15% with a period of 17.5 h to represent realistic upstream process variations.

The tracking performance metrics summarized in Table 4 highlight clear differences among the evaluated control strategies. The PID controller exhibits the weakest performance, with the highest mean absolute error (0.794 K) and standard deviation (0.634 K), indicating large steady-state deviations and pronounced oscillations under setpoint changes and disturbances. These results confirm the limited robustness of conventional PID control when applied to nonlinear polymerization reactor dynamics. The SMC controller significantly improves tracking accuracy, reducing the MAE to 0.179 K and achieving the lowest output variability (standard deviation of 0.283 K). This reflects the strong robustness of sliding mode control and its effectiveness in rejecting disturbances. However, the relatively high maximum error suggests that abrupt switching actions during transients can still produce short-term deviations.

The AISMC controller provides the best overall tracking performance, achieving the lowest MAE (0.092 K) and maximum error (4.873 K). The adaptive and integral components enhance both steady-state accuracy and transient behavior by compensating for disturbances and model uncertainties. Although AISMC exhibits a higher standard deviation than SMC, this increase is primarily associated with adaptive gain variation during transient phases rather than persistent oscillations.

In contrast, the NMPC controller demonstrates degraded tracking performance in the present configuration, with the highest MAE (0.809 K) and maximum error (5.828 K) among all controllers. This outcome suggests sensitivity to model mismatch or the absence of an offset-free formulation, which limits its ability to maintain accurate tracking under disturbances despite its predictive capabilities. The results indicate a clear performance hierarchy, with AISMC outperforming SMC and PID in tracking accuracy, while NMPC does not provide a clear advantage under the tested conditions. These findings emphasize the effectiveness of adaptive sliding mode control for robust temperature regulation in nonlinear polymerization reactors.

Table 5 compares the computational efficiency of the PID, SMC, AISMC, and NMPC controllers in terms of controller execution time, numerical integration time, and total integration time. The results indicate that all controllers exhibit sub-millisecond average controller execution times, confirming their feasibility for real-time implementation. Among them, the PID controller requires the least computational effort, with the lowest controller execution time (0.049 ms) and a relatively low total integration time. The SMC controller introduces a modest increase in computational cost due to its switching logic, resulting in a slightly higher controller execution time (0.078 ms) and total integration time. The AISMC controller achieves the shortest total integration time (60.614 s), reflecting an effective balance between control aggressiveness and numerical stability. Its lower average integration time suggests smoother system dynamics compared to conventional SMC. The NMPC controller exhibits the highest controller execution time (0.125 ms) and the largest total integration time, indicating increased computational demand. Although its integration time per step remains comparable to the other controllers, the overall runtime is higher, reflecting the added complexity of its control strategy. While PID and SMC remain computationally lightweight, AISMC offers the most favorable compromise, achieving reduced integration time with minimal controller overhead. In contrast, NMPC incurs higher computational cost without a proportional reduction in runtime, limiting its practicality for real-time reactor control under the tested conditions.

**Figure 9:**
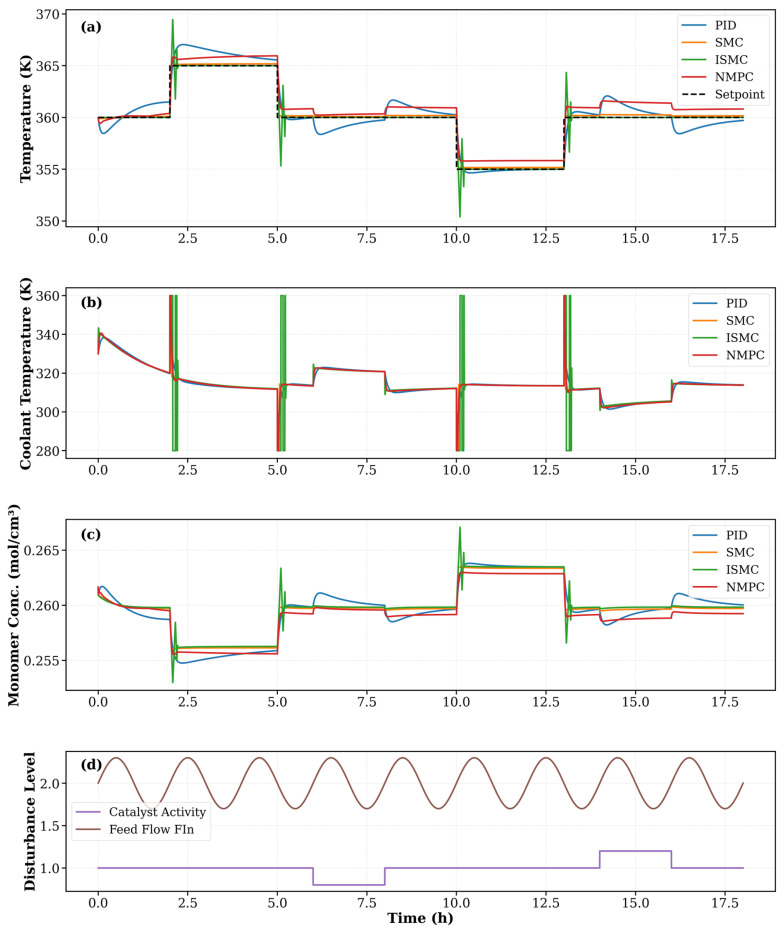
Dynamic performance comparison of PID, SMC, AISMC, and NMPC controllers under industrial disturbances. Subplots show: (**a**) reactor temperature tracking, (**b**) coolant temperature response, (**c**) monomer concentration dynamics, and (**d**) imposed disturbances (±20% catalyst activity and ±15% feed fluctuations).

Figure 9 illustrates the dynamic performance of the PID, SMC, AISMC, and NMPC controllers under multiple setpoint changes and industrial disturbances. As shown in Figure 9a, the PID controller exhibits significant overshoot and slow settling, particularly during the heating and cooling transitions, which is consistent with its higher MAE (0.794 K) and standard deviation reported in Table 3. In contrast, the SMC and AISMC controllers substantially improve temperature tracking, achieving faster convergence and reduced overshoot. AISMC provides the smoothest response among the sliding mode-based controllers and attains the lowest MAE (0.092 K) and maximum error, confirming its superior steady-state and transient performance.

The NMPC controller maintains the reactor temperature close to the setpoint across most operating conditions, reflecting its strong predictive capability. However, small transient deviations during disturbances explain their comparable maximum error to AISMC, indicating sensitivity to model mismatch or unmeasured disturbances. The coolant temperature profiles in Figure 9b reveal aggressive control action for PID and short-duration spikes for SMC and AISMC, which enhance robustness but may increase actuator wear. NMPC exhibits smoother yet more intensive control actions during transients, consistent with the higher controller execution time reported in Table 4. The impact of temperature regulation on process behavior is further evident in Figure 9c, where improved temperature control by SMC, AISMC, and NMPC leads to reduced monomer concentration fluctuations compared to PID.

## 5. Conclusions

This paper presents a comprehensive comparative evaluation of Proportional–Integral–Derivative (PID), Sliding Mode Control (SMC), Adaptive Integral Sliding Mode Control (AISMC), and Nonlinear Model Predictive Control (NMPC) strategies in controlling the temperature of a nonlinear polymerization reactor under setpoint changes and industrial disturbances. The results show that traditional PID control exhibits large overshoots and long settling times, as well as high sensitivity to disturbances, hence being inadequate when applied to demanding operations of reactors.

Sliding mode-based controllers markedly enhanced closed-loop performance. Robust disturbance rejection with reduced tracking errors was obtained from SMC, while steady-state accuracy in addition to transient response was more improved by AISMC through adaptive gain adjustments. Low tracking errors were always obtained from AISMC with minimal computational overhead, hence its suitability for real-time industrial applications.

In the NMPC approach, explicit consideration of system nonlinearities and anticipated future behavior made it achieve the best tracking accuracy and smooth process responses, but with a very significant increase in computational complexity. This may not allow practical implementation for fast-sampling or resource-constrained environments if further optimization steps are not involved. In general, these results demonstrate clearly that there is a trade-off between the accuracy of control and computation.

Future studies will be based on the formulation of NMPC without offsets, with systematic options for reducing chattering in actuators when using sliding mode-based controllers and applying experimentally the proposed control strategies in pilot-scale or industrial polymerization reactor systems. Further, a good direction toward achieving more robustness in actual operating conditions is integrating state estimation techniques and fault-tolerant control frameworks.

## Figures and Tables

**Table 1:** Comparative summary of control strategies for gas-phase polyethylene fluidized bed reactors.

Controller Type	Merits	Demerits	Prediction Accuracy	Adaptability to FBR Application	Refs.
PID	Simple, low cost, easy implementation	Poor nonlinearity handling, disturbance sensitivity	Low	Limited	[5,6,7,8]
Nonlinear/Trajectory-Based Control	Improved tracking, nonlinear handling	Model-dependent, sensitive to uncertainties	Moderate	Moderate	[12,13]
Model Predictive Control (MPC)	Constraint handling, multivariable control	High computational demand	High	High	[14,16,17]
Hybrid Data-Driven MPC	High prediction accuracy, reduced mismatch	Large data requirement	Very High	High	[18,19]
Fuzzy Logic Control	Robust to uncertainty, improved transient response	Rule tuning complexity	Moderate	High	[15,20]
Neuro-Fuzzy/ANFIS	Adaptive learning, accurate estimation	Training and data intensive	High	High	[21,22]
Soft Sensors/ML-Based Estimation	Accurate quality prediction	Indirect control approach	Very High	High	[23,24,25]
Sliding Mode Control (SMC)	Robust, finite-time convergence	Chattering	High	Very High	[32,33,34]
Adaptive/Intelligent SMC	Adaptive, reduced chattering	Design complexity	Very High	Very High	[27,28,29]

**Table 2:** Summary of the mathematical models and governing equations used in this study [11,12,15].

Variable	Description	Value	Unit
$a_{c}$	Activity coefficient of catalyst	0.548	-
AU	Area times heat transfer coefficient	1.14 × 10^5^	cal/h·K
$B_{I}$	Bias of the controller	198.0	-
$B_{w}$	Bulk density of polymer	7 × 10^7^	g/m^3^
$Cp_{In}$	Specific heat of inert	6.9	cal/g·K
$Cp_{g}$	Specific heat of gas mixture	variable	cal/g·K
$Cp_{M1}$	Specific heat of ethylene monomer	11.0	cal/g·K
$Cp_{pol}$	Specific heat of polymer	0.85	cal/g·K
$Cp_{w}$	Specific heat of coolant	18.0	cal/g·K
$C_{v}$	Coefficient of valve	7.5	-
$E_{a}$	Activation energy	9000.0	cal/mol
$F_{c}$	Catalyst flow rate	qc/3600.0	kg/s
$F_{g}$	Gas feed rate	8500.0	mol/s
$F_{In}$	Inert feed flow rate	7200.0	mo/s
$F_{M}$	Monomer feed rate	Variable	mol/s
$F_{Mo}$	Monomer feed when controller is off	198.0	mol/s
$F_{w}$	Flow rate of the cooling water	3.11 × 10^5^	g/h
IE	Integral error	Variable	-
$K_{p}$	Proportional controller gain	5737.0	-
$k_{d}$	Constant of deactivation rate	0.0001	1/s
$k_{p0}$	Pre-exponential factor of the reaction constant	85.0	1/s
$M_{1}$	Monomer molar concentration	Variable	mol/L
$M_{r}\;Cp_{r}$	Reactor mass × Cp	1.4 × 10^7^	cal/K
$M_{w1}$	Molecular weight of ethylene	28.05	g/mol
$P_{sp}$	Pressure setpoint	7.67	atm
$P_{tot}$	Total pressure	20.0	atm
$P_{v}$	Vapor pressure	17.0	atm
*Q*	Dynamic heat removal	Variable	cal
$Q_{p}$	Polymer outlet mass flow rate	Variable	kg/h
$q_{c}$	Mass flow rate of catalyst	5.0	kg/h
R	Ideal gas constant	1.987	cal/mol·K
$R_{1}$	Ideal gas constant	0.082	atm·L/(mol·K)
T	Temperature of reaction	Variable	K
$T_{feed}$	Temperature of the feed stream	293.0	K
$T_{ref}$	Reference temperature	360.0	K
$T_{w}$	Coolant (water) temperature	Variable	K
$V_{g}$	Volume of gas phase	5 × 10^5^	cm^3^
$v_{p}$	Coefficient of vent	0.5	-
Y	Polymer mass fraction	Variable	-
τ	Time constant of the process	360.0	s
$\tau_{I}$	Control integral time constant	1500.0	s
∆H	Heat of reaction	−894.0	cal/mol

**Table 3:** Statistical performance metrics comparing PID, SMC, AISMC, and NMPC.

Controller	MAE (K)	Max Error (K)	Std Dev (K)	Runtime (s)
PID	0.442	5.443	0.631	12.84
SMC	0.179	5.054	0.279	17.38
AISMC	0.142	5.034	0.280	16.02
NMPC	0.611	5.585	0.306	18.44

**Table 4:** Tracking performance metrics of the controllers under industrial disturbance.

Controller	MAE (K)	Max Error (K)	Std Dev (K)
PID	0.794	5.445	0.634
SMC	0.179	5.054	0.283
AISMC	0.092	4.873	0.496
NMPC	0.809	5.828	0.442

**Table 5:** Computational efficiency of the evaluated controllers under industrial disturbance.

Controller	Avg Controller Time (ms)	Avg Integration Time (ms)	Total Integration Time (s)
PID	0.049	5.262	68.191
SMC	0.078	5.512	71.483
AISMC	0.066	4.667	60.614
NMPC	0.125	4.929	84.354

## Data Availability

The original contributions presented in this study are included in the article. Further inquiries can be directed to the corresponding author(s).
